# The Role of Psychosocial Factors for Depressive Symptoms in Caregivers of People with Dementia

**DOI:** 10.1002/alz70858_102570

**Published:** 2025-12-25

**Authors:** Lina Charlotte Jeran, Nadia Blecha, Jochen René Thyrian, Wolfgang Hoffmann, Iris Blotenberg

**Affiliations:** ^1^ Deutsches Zentrum für Neurodegenerative Erkrankungen e. V. (DZNE), site Rostock / Greifswald, Greifswald, Mecklenburg‐Vorpommern, Germany; ^2^ Institute for Community Medicine, University Medicine Greifswald, Greifswald, Mecklenburg‐Vorpommern, Germany

## Abstract

**Background:**

Taking care of a person with dementia is challenging and can contribute to depressive symptoms in caregivers. To provide tailored support, predictors of depressive symptoms need to be better understood. Yet, there is a lack of longitudinal studies and little knowledge about the role of psychosocial factors. To address this gap, this study investigates the role of perceived lack of appreciation as well as perceived burden of losing the relationship to the person with dementia on depressive symptoms in caregivers longitudinally – beyond known demographic and clinical predictors.

**Method:**

We analyzed data from 179 dyads consisting of people with dementia (Mage = 80, 61.5% female) and their caregivers (Mage = 64.5, 70.9% female) in Germany. Dyads were interviewed annually by specially qualified nurses over a time span of four years. Depressive symptoms of caregivers were assessed with the Patient Health Questionnaire (PHQ), psychosocial factors were assessed using the Berlin Inventory of Caregiver Burden (BIZA‐D). We employed a multilevel growth curve model with random intercepts and slopes. We investigated the role of both within‐ and between‐person differences in psychosocial factors for depressive symptoms in caregivers.

**Result:**

Perceived lack of appreciation and the perceived burden of losing the relationship to the person with dementia were significant both as within‐ and between‐person differences. The severity of cognitive symptoms in the person with dementia was significant as between‐ but not within‐person difference. Neuropsychiatric symptoms were significant as within‐ but not between‐person difference.

**Conclusion:**

For the first time, this study indicates that psychosocial factors play an important role in the development of depressive symptoms in caregivers of people with dementia. Appreciation by the social environment and addressing the changing relationship between the caregiver and the person with dementia might be promising targets for interventions.

